# Clinical Analysis of Preoperative Anti-thyroglobulin Antibody in Papillary Thyroid Cancer Between 2011 and 2015 in Beijing, China: A Retrospective Study

**DOI:** 10.3389/fendo.2020.00452

**Published:** 2020-07-15

**Authors:** Xiaomeng Jia, Ping Pang, Lin Wang, Ling Zhao, Lina Jiang, Yeqiong Song, Xiaojing Fan, Yajing Wang, Sitong Zhao, Jianming Ba, Guoqing Yang, Xianling Wang, Weijun Gu, Li Zang, Yu Pei, Jin Du, Yiming Mu, Zhaohui Lyu

**Affiliations:** ^1^Department of Endocrinology, Chinese PLA General Hospital, Beijing, China; ^2^Department of Endocrinology, Hainan Branch of PLA General Hospital, Sanya, China; ^3^Department of Internal Medicine, No. 316 Hospital of PLA, Beijing, China; ^4^The People's Liberation Army Troop, Zhangjiakou, China; ^5^Department of Endocrinology, The Third People's Hospital, Hangzhou, China

**Keywords:** thyroglobulin antibodies, papillary thyroid cancer, thyroid nodule, thyroidectomy, lymph node metastasis

## Abstract

The anti-thyroglobulin antibody (TgAb) has been suggested to be more common in patients with papillary thyroid cancer (PTC). Here, we performed a retrospective study investigated the correlation between TgAb level and PTC in Chinese patients between 2011 and 2015. Patients with goiter who underwent thyroidectomy and received a confirmed pathological diagnosis were enrolled into the study. Clinical characteristics and preoperative thyroglobulin antibody (TgAb) level data were collected from all enrolled patients. Based on the preoperative TgAb test results, patients were divided into a TgAb negative (TgAb–) group (<60 IU/mL) and a TgAb positive (TgAb+) group (≧60 IU/mL). Of the 4,046 patients, 671 patients were TgAb+ while 3,375 patients were TgAb–. There were 535 (79.7%) patients with PTC in the TgAb+ group, and 2,154 (63.8%) patients with PTC in the TgAb– group. The prevalance of PTC was significantly higher in TgAb+ patients than in TgAb– patients. TgAb+ patients were stratified into four groups based on the TgAb titer. The prevalence of PTC did not increase with TgAb titer. No significant difference in TgAb level was noted in patients with different clinicopathologies, including TNM stage, lymph node metastasis, and multifocal carcinoma. Regression analysis suggested a higher risk of PTC malignancy among TgAb+ patients. Preoperative TgAb level ≥60 IU/mL might be associated with a higher risk of PTC. However, there was no titer-dependent association between elevated TgAb titer and PTC malignancy.

## Introduction

Thyroid nodules are a common cinical problem. The purpose of regular tests like ultrasonography or fine needle aspiration is to determine whether the nodules are cancerous, which occurs in 7–15% of cases. This rate varies with age, gender, radiation exposure history, and other factors (1). Differentiated thyroid cancer (DTC) accounts for ~90% of thyroid cancers, with papillary thyroid carcinoma (PTC) as the most common subtype. Effective clinical assessment of thyroid nodules would potentially be able to provide better guidance in the diagnosis and treatment of patients, and could also enable timely intervention to be made in personalized therapy. In fact, DTC prognostics has indeed been an area of intense focus in the field of cancer research. Thyroglobulin (Tg) is widely recognized as a sensitive biomarker of recurrent or residual disease in DTC (2–5). However, the predictive efficacy of Tg may be influenced by the presence of the thyroglobulin antibody (TgAb) (6, 7). In China, the TgAb test is significantly more common than the Tg test, especially in remote areas. In general, patients with DTC show higher TgAb levels than those without DTC (8, 9), suggesting a potential predictive role for TgAb in DTC. Patients with a high postoperative level of TgAb may be at risk of DTC recurrence or having residual tumor after surgery. Thus, the postoperative TgAb level could be a useful marker for DTC cancer recurrence prognosis (10). However, the association between preoperative TgAb and DTC is still controversial. The TgAb is generally detected in patients with autoimmune thyroid disease (AITD) (11–13), which accounts for a higher prevalence of DTC and is thought to be related to more favorable tumor-node-metastasis staging (13, 14). Indeed, almost 50% of TgAb+ patients have been diagnosed with chronic lymphocytic thyroiditis, indicating the role of autoimmunity in the association between TgAb and DTC. Recognition of different epitopes on Tg by TgAb has been found to underlie the progression of HT and DTC, indicating the presence of distinct mechanisms of TgAb generation in these two diseases (15, 16). Therefore, in this study, we investigated the association of preoperative TgAb status and PTC as well as exploring their relationship with HT. In addition, we also examined the titer-dependent effect and prognostic value of preoperative TgAb status in PTC.

## Materials and Methods

### Clinical Data

**Figure 1:**
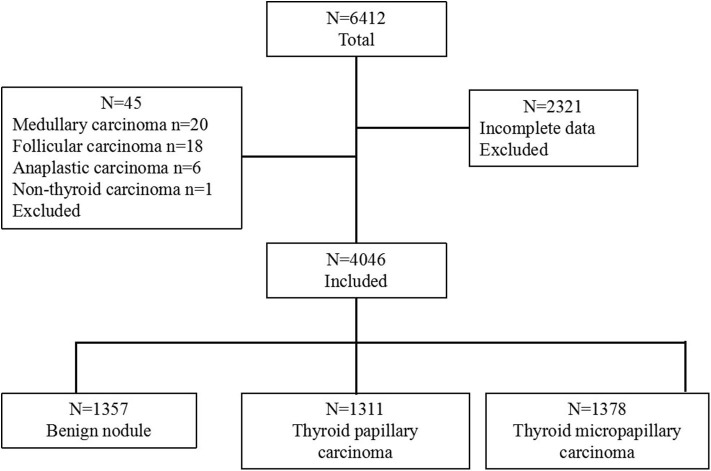
Flow chart showing the categorization of 6,412 patients into 3 groups based on diagnosis. The group with 4,046 patients was further categorized into 3 pathological groups: benign nodule, thyroid papillary carcinoma, and thyroid micropapillary carcinoma.

Between January 2011 and December 2015, 6,412 patients with thyroid nodules who underwent thyroidectomy at the Department of General Surgery in PLA General Hospital, Beijing, China, were enrolled in this retrospective study. Among them, 2,314 patients without preoperative TgAb test results were excluded. In addition, 45 cases with other types of carcinoma (medullary carcinoma, follicular carcinoma, anaplastic carcinoma, and malignant lymphoma carcinoma) were also excluded from the present study. Considering the exclusions, only 1,357 benign cases and 2,889 PTC cases with a clear pathological diagnosis and complete medical records were retrospectively reviewed (Figure 1). The study was approved by the local Ethical Committee of the People's Liberation Army General Hospital, Beijing, China. Written informed consent was obtained from all patients after a full explanation of the purpose and procedures to be performed in this study.

### Patient Evaluation and Laboratory Tests

All patients underwent a thyroid ultrasound (US) examination 1 month before surgery. Patients with suspicious US features were subjected to fine-needle aspiration cytology (FNAC). Patients that required surgery were diagnosed according to surgical indications, wherein more than one thyroid nodule with suspicious US features were detected and confirmed by ultrasound. Suspicious US features include hypoechogenicity, microcalcifications, infiltrative margins, anteroposterior/transversal (AP/TR) diameter ≥1, and large nodules with clinical findings indicating malignancy or indeterminate/suspicious FNAC finding. Biochemical tests were performed between 1 day and 1 week before the operation. The levels of TPOAb and TgAb were detected using an anti-TPO kit and an anti-Tg kit (SIEMENS, USA) with the same automated immune chemiluminescent assay used at the central laboratory of the hospital. A value of <60 IU/mL was considered a negative result. TgAb+ patients were subjected to quartile stratification [Group1 (control): 60–100.8 IU/mL; Group2: 100.9–159.8 IU/mL; Group3: 159.9–272.6 IU/mL; Group4: >272.6 IU/mL] for the analysis of the relationship between TgAb titer and PTC malignancy. Serum TSH (normal range of 0.35–5.5 mU/L) was detected using a TSH3-Ultra immunoassay kit (SIEMENS, USA). The sensitivity of TSH assay was 0.01 mIU/L. Serum TSH values were classified as follows: ≦0.3, 0.3–1.0, 1.0–1.9, 1.9–4.8, and >4.8 mIU/L, based on cutoff values that were predetermined in a previous population study (17).

### Pathological Diagnosis

Postoperative pathological results were identified by an experienced pathologist, to determine the pathological type of the nodules. The largest tumor size was measured in patients with multifocal PTC. All the malignant patients were graded based on the staging system suggested by the American Joint Committee on Cancer. Hashimoto's thyroiditis (HT) was diagnosed based on the following histological criteria: (1) diffuse infiltration of lymphocyte and plasma cells, germinal center; (2) lymphoid follicles with germinal centers; (3) parenchyma atrophy with eosinophilic change; and interstitial fibrosis.

### Statistical Analysis

The data were analyzed using SPSS 20.0 (SPSS Corp, Chicago, USA). Data comparison was performed using a *t*-test and data were categorized by the Chi-square test or Fisher's exact test, assuming a normal distribution. Multivariate logistic regression analysis was performed to evaluate the association between PTC malignancy and risk factors, such as age, sex, nodule size, TSH, HT, and TgAb positivity. A value of *P* < 0.05 was considered statistically significant.

## Results

### General Characteristics of Patients Diagnosed With Benign Nodule and PTC

Of the 4,046 goiter patients, 2,885 were female and 1,161 were male (female-to-male ratio of 5:2), and the mean age was 47.92 ± 11.391 years old (age ranging from 18 to 85 years old). The benign nodule (BN) group showed TgAb positivity in 136 out of 1,357 patients (10.0%), which was around 2-fold lower (*P* < 0.05) than that for the PTC group, which was 535 out of 2,689 patients (19.9%). In female patients, the PTC group showed higher levels of TgAb positivity than those observed in the BN group (12.4 vs. 25.1%; *P* < 0.001). A similar trend was observed in male patients, with TgAb positivity in 3.4% of the BN group and 7.7% of the PTC group (*P* < 0.05). Regardless of gender, patients with PTC were younger in age, had smaller tumor size, higher prevalence of HT and higher TSH levels. However, the distribution of TPOAb positivity showed no significant difference between the BN and PTC groups (Table 1).

**Table 1:** Characteristics of patients with benign nodule and PTC in different genders.

**Variables**	**Total**			**Male**			**Female**		
	**Benign nodule *N* = 1,357**	**PTC *N* = 2,689**	***P*-value**	**Benign nodule *N* = 357**	**PTC *N* = 804**	***P*-value**	**Benign nodule *N* = 1,000**	**PTC *N* = 1,885**	***P*-value**
Age, mean ± SD, years	50.35 ± 11.66	44.36 ± 10.70	<0.001	51.46 ± 10.54	43.50 ± 10.99	<0.001	49.96 ± 12.02	44.72 ± 10.56	<0.001
Nodule size, mean ± SD, cm	3.12 ± 1.77	1.11 ± 0.80	<0.001	3.13 ± 1.75	1.22 ± 0.89	<0.001	3.13 ± 1.78	1.07 ± 0.76	<0.001
HT [*n*(%)]	70 (5.2)	279 (10.4)	<0.001	9 (2.5)	26 (3.2)	0.512	61 (6.1)	253 (13.4)	<0.001
TSH, median (IQR), mIU/L	1.65 (0.94–2.64)	1.98 (1.29–2.97)	<0.001	1.55 (0.90–2.31)	1.65 (1.09–2.46)	<0.001	1.72 (0.97–2.80)	2.14 (1.42–3.24)	<0.001
TPOAb+ [*n*(%)]	202 (14.9)	458 (17.0)	0.081	48 (13.4)	91 (11.3)	0.303	154 (15.4)	367 (19.5)	0.007
TgAb+ [*n*(%)]	136 (10.0)	535 (19.9)	<0.001	12 (3.4)	62 (7.7)	0.008	124 (12.4)	473 (25.1)	<0.001
Radiation history of neck [*n*(%)]	3 (0.2)	13 (0.5)	0.402	0 (0.0)	4 (0.5)	0.377	3 (0.3)	13 (0.7)	0.406
Family history of TC [*n*(%)]	3 (0.2)	16 (0.6)	0.247	0 (0.0)	2 (0.3)	0.533	3 (0.3)	11 (0.6)	0.481

*TgAb–, negative preoperativeTgAb; TgAb+, positive preoperative TgAb; HT, Hashimoto's thyroiditis; TSH, serum thyroid stimulating hormone. Radiation history of neck: Radiation history of neck in children and adolescence*.

### Correlation Between Preoperative TgAb Status and PTC

Based on the TgAb titer, patients were divided into TgAb+ and TgAb– groups. Among 4,046 patients, 671 patients were TgAb+ while 3,375 patients were TgAb–. According to the diameter of the largest tumor, malignant nodules were defined as either microcarcinoma (<1 cm) or larger carcinoma (≥1 cm). In the TgAb+ group, 136 (20.3%) patients were diagnosed with benign nodules, 272 (40.5%) with microcarcinoma and 263 (39.2%) with larger carcinoma. In TgAb– patients, 1,221 (36.2%) patients were diagnosed with BN, and 2,154 patients with PTC, of which 1,106 (32.8%) had microcarcinoma and 1,048 (31.1%) had larger carcinoma. The prevalence of malignancy was significantly higher in TgAb+ patients than in TgAb– patients (Table 2). Logistic regression analysis, with and without adjustment for age, larger nodule size, serum TSH and TPOAb, suggested the positive correlation between TgAb and PTC [including papillary thyroid microcarcinoma (PTMC)] in both male and female patients (Table 3).

**Table 2:** Characteristics of male and female TgAb-negative and TgAb-positive patients.

**Variables**	**Total**			**Male**			**Female**		
	**TgAb– *N* = 3,375**	**TgAb+ *N* = 671**	***P*-value**	**TgAb– *N* = 1,087**	**TgAb+ *N* = 74**	***P*-value**	**TgAb– *N* = 2,288**	**TgAb+ *N* = 597**	***P*-value**
Age, mean ± SD, years	46.52 ± 11.39	45.58 ± 11.39	0.049	45.98 ± 11.34	45.38 ± 13.03	0.661	46.78 ± 11.40	45.60 ± 11.18	0.024
Nodule size, mean ± SD, cm	1.85 ± 1.57	1.46 ± 1.36	<0.001	1.84 ± 1.52	1.37 ± 1.05	<0.001	1.86 ± 1.59	1.47 ± 1.39	<0.001
HT [*n*(%)]	94 (2.8)	255 (38.0)	<0.001	11 (1.0)	24 (32.4)	<0.001	83 (3.6)	231 (38.7)	<0.001
TSH, median(IQR), mIU/L	1.83 (1.14–2.79)	2.10 (1.42–3.34)	<0.001	1.62)	1.95 (1.28–2.85)	0.032	1.97 (1.21–3.04)	2.17 (1.43–3.42)	<0.001
TPOAb+ [*n*(%)]	358 (10.6)	302 (45.0)	<0.001	101 (9.3)	38 (51.4)	<0.001	257 (11.2)	264 (44.2)	<0.001
Pathological diagnosis			<0.001			0.019			<0.001
Benign nodule [*n*(%)]	1,221 (36.2)	136 (20.3)		345 (31.7)	12 (16.2)		876 (38.3)	124 (20.8)	
PTMC [*n*(%)]	1,106 (32.8)	272 (40.5)		350 (32.2)	30 (40.5)		756 (33.0)	242 (40.5)	
PTC [*n*(%)]	1,048 (31.1)	263 (39.2)		392 (36.1)	32 (43.2)		656 (28.7)	231 (38.7)	

**Table 3:** Logistic regression analysis to determine the associations between tested factors and TgAb status in different genders.

**Variables**	**Unadjusted**		**Adjusted**	
	**OR (95% CI)**	***P*-value**	**OR (95% CI)**	***P*-value**
**Total**				
Age, years	0.993 (0.985–1.000)	0.049	1.004 (0.995–1.013)	0.344
Male	0.261 (0.230–0.336)	<0.001	0.311 (0.235–0.412)	<0.001
Nodule size, cm	0.821 (0.769–0.876)	<0.001	0.989 (0.897–1.091)	0.823
**TSH, mIU/L**				
<0.3	1.000		1.000	
0.3–1.0	0.849 (0.531–1.357)	0.494	1.224 (0.706–2.122)	0.471
1.0–1.9	0.901 (0.585–1.388)	0.636	1.029 (0.617–1.717)	0.913
1.9–4.8	1.226 (0.805–1.867)	0.343	1.144 (0.693–1.887)	0.600
≥4.8	1.954 (1.211–3.152)	0.006	0.996 (0.558–1.776)	0.988
TPOAb+	6.897 (5.718–8.319)	<0.001	4.660 (3.744–5.801)	<0.001
**Pathological diagnosis**				
Benign nodule	1.000		1.000	
PTMC	2.208 (1.770–2.755)	<0.001	2.104 (1.478–2.993)	<0.001
PTC	2.253 (1.803–2.816)	<0.001	2.207 (1.639–2.974)	<0.001
**Female**				
Age, years	0.991 (0.983–0.999)	0.024	1.002 (0.992–1.012)	0.683
Nodule size, cm	0.828 (0.773–0.888)	<0.001	0.999 (0.901–1.108)	0.985
**TSH, mIU/L**				
<0.3	1.000		1.000	
0.3–1.0	1.036 (0.623–1.723)	0.891	1.282 (0.710–2.316)	0.410
1.0–1.9	1.049 (0.658–1.671)	0.841	1.142 (0.661–1.973)	0.635
1.9–4.8	1.284 (0.817–2.019)	0.279	1.213 (0.711–2.070)	0.479
≥4.8	1.686 (1.010–2.813)	0.046	1.010 (0.548–1.862)	0.975
TPOAb+	6.265 (5.093–7.708)	<0.001	5.285 (3.384–5.427)	<0.001
**Pathological diagnosis**				
Benign nodule	1.000		1.000	
PTMC	2.261 (1.784–2.867)	<0.001	2.044 (1.407–2.970)	<0.001
PTC	2.488 (1.956–3.164)	<0.001	2.155 (1.569–2.960)	<0.001
**Male**				
Age, years	0.995 (0.975–1.016)	0.660	1.012 (0.987–1.038)	0.348
Nodule size, cm	0.761 (0.618–0.936)	0.010	0.961 (0.707–1.307)	0.800
**TSH, mIU/L**				
<0.3	1.000		1.000	
0.3–1.0	0.702 (0.187–2.629)	0.599	0.943 (0.231–3.848)	0.935
1.0–1.9	0.710 (0.204–2.477)	0.591	0.512 (0.133–1.976)	0.331
1.9–4.8	1.013 (0.296–3.467)	0.984	0.749 (0.197–2.850)	0.672
≥4.8	3.556 (0.869–14.546)	0.078	1.143 (0.205–6.389)	0.879
TPOAb+	10.305 (6.252–16.985)	<0.001	7.496 (4.165–13.490)	<0.001
**Pathological diagnosis**				
Benign nodule	1.000		1.000	
PTMC	2.464 (1.241–4.892)	0.010	3.356 (1.117–10.085)	0.031
PTC	2.347 (1.190–4.628)	0.014	3.433 (1.385–8.510)	0.008

*Multivariate-adjusted model included age; gender (among total patients); Nodule size (largest tumor size); TSH (thyroid stimulating hormone) (uIU/l); TPOAb+ (positive preoperative thyroid peroxidase antibody); Pathological diagnosis (benign nodule, papillary thyroid microcarcinoma, papillary thyroid carcinoma)*.

### The Prevalence of PTC in TgAb-Stratified Groups

**Figure 2:**
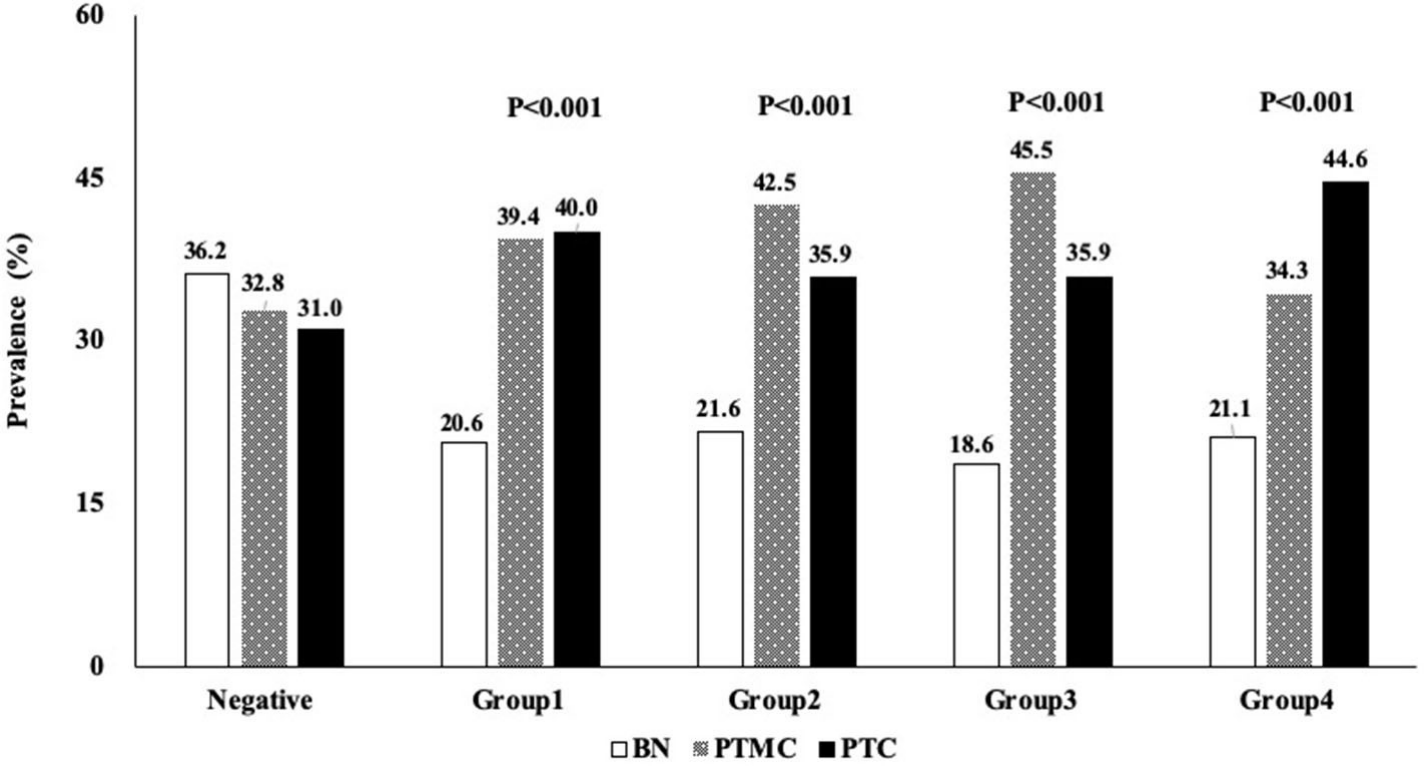
Profile showing the percentage distribution of 4 groups of patients with different serum TgAb titer who were diagnosed with BN, PTMC, or PTC. BN, benign; PTC, papillary thyroid cancer; PTMC, papillary thyroid microcarcinoma; TgAb, anti-thyroglobulin antibody.

TgAb+ patients were divided into four groups according to TgAb titer by quartile stratification [Group1 (60–100.8 IU/mL), Group2 (100.9–159.8 IU/mL), Group3 (159.9–272.6 IU/mL), Group4 (>272.6 IU/mL)] (Figure 2). TgAb+ patients showed higher prevalence of PTMC and PTC in all 4 subgroups [Group1 (39.4, 40.0%; *P* < 0.001), Group2 (42.5, 35.9%; *P* < 0.001), Group3 (45.5, 35.9%; *P* < 0.001), Group4 (34.3, 44.6%; *P* < 0.001)]compared to TgAb– patients (32.8; 31.0%). No significant difference in malignancy prevalence was noted between the four subgroups (Figure 2).

### Logistic Regression Analysis of the Related Factors of PTC Malignancy

In the multivariate model, the OR of positive preoperative TgAb was 2.230 (1.824–2.726) After adjustment for age, gender, largest nodule size, HT, and serum TSH, the results still suggested positive correlation between preoperative TgAb and PTC, with an OR of 2.012 (1.497–2.705). The titer-dependent analysis indicated that compared with negative group (preoperative TgAb<60 IU/mL), the ORs of Group1 (TgAb: 60–100.8 IU/mL), Group2 (TgAb: 100.9–159.8 IU/mL), Group3 (TgAb: 159.9–272.6 IU/mL), and Group4 (TgAb: >272.6 IU/mL) were 2.183 (95% CI 1.487–3.204), 2.062 (95% CI 1.416–3.001), 2.486 (95% CI 1.672–3.695), and 2.121 (95% CI 1.451–3.099), respectively (Table 4).

**Table 4:** Logistic regression analysis to determine the association between tested factors and PTC.

**Variables**	**Unadjusted**		**Adjusted**	
	**OR (95% CI)**	***P*-value**	**OR (95% CI)**	***P*-value**
Age, years	0.952 (0.946–0.958)	<0.001	0.962 (0.955–0.970)	<0.001
Male	0.967 (0.832–1.126)	0.671	0.842 (0.821–1.049)	0.587
Nodule size, cm	0.266 (0.244–0.289)	<0.001	0.272 (0.249–0.296)	<0.001
HT	2.128 (1.624–2.790)	<0.001	0.959 (0.658–1.399)	0.829
**TSH, mIU/L**				
<0.3	1.000		1.000	
0.3–1.0	1.003 (0.719–1.399)	0.986	0.685 (0.422–1.113)	0.127
1.0–1.9	1.972 (1.442–2.696)	<0.001	1.084 (0.687–1.710)	0.728
1.9–4.8	2.319 (1.703–3.157)	<0.001	1.245 (0.794–1.952)	0.339
≥4.8	1.989 (1.362–2.905)	<0.001	1.272 (0.744–2.174)	0.379
TPOAb+,	1.174 (0.980–1.406)	0.081	0.908 (0.700–1.179)	0.470
TgAb+	2.230 (1.824–2.726)	<0.001	2.012 (1.497–2.705)	<0.001
**TgAb stratification**				
Negative	1.000		1.000	
Group1	2.183 (1.487–3.204)	<0.001	2.336 (1.578–3.457)	<0.001
Group2	2.062 (1.416–3.001)	<0.001	2.350 (1.595–3.461)	<0.001
Group3	2.486 (1.672–3.695)	<0.001	2.753 (1.831–4.139)	<0.001
Group4	2.121 (1.451–3.099)	<0.001	2.296 (1.549–3.403)	<0.001

*HT was diagnosed by histological analysis*.

*Multivariate-adjusted model included age; gender; Nodule size (largest tumor size); HT, Hashimoto's thyroiditis; TSH, thyroid stimulating hormone (uIU/l); TPOAb+, positive preoperative thyroid peroxidase antibody; TgAb+, positive preoperative thyroglobulin antibody; TgAb stratification, Negative TgAb <60 IU/mL; Group1 TgAb: 60–100.8 IU/mL, Group2 TgAb: 100.9–159.8 IU/mL, Group3 TgAb: 159.9–272.6 IU/mL, Group4 TgAb: >272.6 IU/mL*.

### TgAb and Clinicopathological Features of Patients With PTC

Of the 2,689 patients with PTC, 2,154 patients were TgAb– and 535 patients were TgAb+. We evaluated the pathological features in patients with different TgAb levels. There was no obvious difference in the proportion of patients with PTMC, TNM stage I/II, lymph node metastasis and multifocal carcinoma (Table 5).

**Table 5:** Clinical features of TgAb-negative and -positive patients with PTC.

**Factor**	**Total**		
	**TgAb– *N* = 1,675**	**TgAb+ *N* = 426**	***P*-value**
Age, mean ± SD, years	44.38 ± 10.64	44.25 ± 10.97	0.161
**Gender [*****n*****(%)]**			
Male	1,087 (32.3)	74 (11.0)	<0.001
Female	2,288 (67.9)	597 (89.0)	
Nodule size, mean ± SD, cm	1.12 ± 0.82	1.09 ± 0.74	0.082
HT [*n*(%)]	79 (3.7)	200 (37.4)	<0.001
TSH, median(IQR), mIU/L	1.95 (1.26–2.89)	2.16 (1.47–3.33)	<0.001
TPOAb+ [*n*(%)]	216 (10.0)	242 (45.2)	<0.001
**Pathological diagnosis [*****n*****(%)]**			
PTMC	1,106 (51.3)	272 (50.8)	0.834
PTC	1,048 (48.7)	263 (49.2)	
TNM stage I/II [*n*(%)]	1,875 (87.0)	461 (86.1)	0.917
Lymph node metastasis [*n*(%)]	555 (25.8)	147 (27.5)	0.420
Multifocal [*n*(%)]	1,095 (65.4)	291 (68.3)	0.253

## Discussion

Thyroid cancer, the most common endocrine carcinoma, has shown a sharp increase in incidence rate within the past few decades (18). Thus, extensive research has been directed toward the screening and identification of reliable predictive factors of thyroid cancer. Notably, the potential predictive factors, thyroglobulin (Tg), and its antibodies, has attracted considerable attention. Although Tg is an indicator of recurrent or metastatic DTC, a higher level of preoperative serum Tg does not necessarily correlate with malignancy arising from the susceptibility to factors such as thyroid mass or TSH stimulation (19). As a matter of fact, the available immunometric assay methods cannot detect Tg bound with TgAb, and that might influence the predictive efficiency of Tg. In China, the TgAb test is much more common than the Tg test. Thus, motivating the need to determine and clarify the clinical significance of TgAb status as a prognostic marker for the risk of thyroid nodule occurrence. There is currently no consensus on the role of preoperative TgAb in PTC surveillance (10, 20–22). Moreover, the relationship between TgAb titer and PTC prevalence has rarely been explored. In the present study, we report a higher prevalence of PTC in patients showing preoperative TgAb positivity. However, a further increase in serum preoperative TgAb titer was not correlated with the rising prevalence of PTC. Although the correlation between TgAb and lymph node metastasis in DTC has also been proposed by some previous studies (23–26), our results show that the prevalence of lymph node metastasis appeared to be slightly higher in patients with PTC that are TgAb+ than those that are TgAb–. However, this difference was not statistically significant.

In the present study, TgAb was regarded as a marker for AITD. This marker is usually used to diagnose HT. Hence, evaluating the role of HT when investigating the relationship between TgAb and PTC is inevitable. Our results indicate that the prevalence of HT in patients with PTC was significantly higher than in those with benign nodules. Regression analysis also showed that HT was correlated with PTC. We next examined whether HT influences TgAb status in patients with PTC. After adjusting for gender, age, serum thyrotropin, and preoperative TgAb, the results were not statistically significant, indicating that the correlation between TgAb and PTC is independent of HT. Consistent with our results, in 2010, Kim et al. reported for the first time that positive serum TgAb is an independent predictive marker for thyroid malignancy in patients diagnosed with thyroid nodules, regardless of the presence of autoimmune thyroiditis (AIT) (10). Recent studies by Hosseini and Grani have also suggested the use of preoperative TgAb levels as a marker for well-differentiated thyroid cancer (21, 22). Although multiple studies have reported a correlation between AITD and DTC, this relationship is still controversial (27–29). It appears that the diagnostic method used influences the association between HT and DTC. Unlike studies using thyroidectomy, studies using fine needle aspiration cytology (FNAC) to examine the relationship between HT and DTC tend not to show a higher risk of DTC (30, 31). In addition, there are few studies that document HT diagnosis, making it difficult to determine whether patients have HT before the occurrence of thyroid carcinoma. Therefore, the causal relationship between HT and DTC is not clear and it is uncertain whether TgAb is generated by the same pathological process in both HT and PTC. Okosieme's study in 2005 identified the epitopes on Tg that are recognized by TgAb-positive sera from patients with DTC. The results suggested that 58% of patients with DTC showed restricted epitope heterogeneity, preferentially recognizing the immune-dominant clusters I, IV, and III in a pattern similar to that seen with typical AITD, while 42% of patients reacted with epitopes in a broadly heterogeneous pattern (32). In the follow-up study by Latrofa, the patients were futher expanded [AITD, non-AITD, HT, Grave's disease (GD), PTC, PTC with thyroid lymphocytic infiltration (PTC-T), and non-toxic multinodular goiter (NTMG)] (15). They found that the pattern of Tg recognition differs from AITD and non-AITD patients. The Tg epitopic regions in non-AITD patients were more variable than those in AITD patients. Moreover, the pattern of recognition of TgAb from PTC-T patients is more similar to that observed in HT than that observed in patients with PTC, which clearly supports the hypothesis that, in patients with PTC, the pattern of TgAb epitopes in quite different from that observed in patients with HT (15)^.^ Other DTC-focused research also made similar conclusions (16).

Several studies have reported that the correlation between TgAb and the risk of PTC was due to the elevated TSH levels observed in some patients AITD who developed hypothyroidism (30, 33–38). An increase in the level of TSH may have stimulated follicular epithelial proliferation, which in turns promotes the development of papillary carcinoma. In our study, the preoperative serum TSH levels were significantly higher in patients with PTC compared to the levels observed in patients with benign nodules (1.98 vs. 1.65 mIU/L, *P* < 0.001). The results of logistic regression modeling showed that the OR for DTC increased along with an increasing serum TSH level for serum TSH<4.8 mIU/L. After adjusting for HT, TgAb, age, and gender, the ORs of TSH in each classified subgroups were reduced, and no significant differences were found. This finding was in contrast to the results of unadjusted anlaysis. Thus, the present study supported the role of preoperative TgAb as a related factor of PTC that is independent of TSH and also the presence of HT, evident from the highest adjusted OR among related factors. According to a systematic review of TSH and thyroid cancer research, studies accounting for autoimmunity have a markedly attenuated OR for the TSH-thyroid cancer relationship. While studies that adjust for age and gender (without adjustment for AITD) have a much higher OR than those that adjust for thyroid autoimmunity. This implies that age and gender might introduce ascertainment bias into the investigation of the association between TSH and thyroid carcinoma, and HT and thyroid carcinoma (39). Consequently, the current study was not able to sufficiently prove the relationship between autoimmunity, TSH and PTC.

In our study, patients in the PTC group were significantly younger than those in the benign group. These results were consistent with a higher detection rate of thyroid nodules with age, reflecting an improvement in the results of the consciousness of health examination. Older patients with suspicious thyroid nodules are potentially more reluctant to receive surgical treatment, considering the higher risks associated with anesthesia or other cardiovascular and cerebrovascular diseases. Our results suggest that a smaller nodule size is correlated with an increased risk of developing PTC. These results were consistent with those reported in our previous study, which showed a significant decrease in the size of thyroid nodules, from a diameter of 2.6 ± 1.4 cm to 1.2 ± 0.9 cm, in patients who underwent thyroidectomy between 1994 and 2015 (40). This trend correlates with the increase in the rate of preoperative fine-needle aspiration cytology, which ranged from 15% in 2004 to 74% in 2013 (40).

In conclusion, this study reports an association between TgAb positivity and the risk of developing PTC, and suggests that clinicians should take this into consideration when determining PTC prognosis in patients with elevated TgAb levels. Future research should be directed toward clarifying the feasibility of using TgAb status as a predictive factor in PTC.

## Data Availability Statement

The datasets generated for this study are available on request to the corresponding author.

## Ethics Statement

The studies involving human participants were reviewed and approved by the local Ethical Committee of the People's Liberation Army General Hospital, Beijing, China. The patients/participants provided their written informed consent to participate in this study.

## Author Contributions

XJ, PP, and ZL designed the study. XJ, PP, LW, LZh, LJ, YS, XF, YW, and SZ collected and analyzed the data. JB, YM, GY, XW, WG, LZa, YP, and JD contributed samples for this study and provided intellectual input. XJ drafted and wrote the manuscript. PP, LW, YM, and ZL performed critical revisions to the manuscript. All authors gave intellectual input to the study and approved the final version of the manuscript.

## Conflict of Interest

The authors declare that the research was conducted in the absence of any commercial or financial relationships that could be construed as a potential conflict of interest.
